# Metagenomic insights into the rumen epithelial integrity responses to the vitamin B1 supplement under high-concentrate diets treatments

**DOI:** 10.3389/fmicb.2022.1008373

**Published:** 2022-10-25

**Authors:** Peng Ma, Chaoqun Sun, Minze Liu, Hongnan You, Yao Shen, Yajie Kang, Yuqin Sun, Zhengang Yang, Pengyun Ma, Liang Yang, Fuguang Xue

**Affiliations:** ^1^Nanchang Key Laboratory of Animal Health and Safety Production, Jiangxi Agricultural University, Nanchang, Jiangxi, China; ^2^State Key Laboratory of Animal Nutrition, Institute of Animal Science, Chinese Academy of Agricultural Sciences, Beijing, China; ^3^Yangxin Yiliyuan Halal Meat Co., Ltd., Yangxin, Shandong, China; ^4^School of Foreign Language, Jiangxi Agricultural University, Nanchang, Jiangxi, China; ^5^Anyou Biotechnology Group Co., Ltd., Taicang, Jiangsu, China

**Keywords:** vitamin B1, high-concentrate diets, rumen fermentability, rumen epithelium, rumen microbiome

## Abstract

Subacute ruminal acidosis (SARA) becomes the most common nutritional metabolic disease in high-yielding dairy cows and later fatting beef cattle because of the increasing consumption of high-concentrate diets in modern feeding patterns. Our previous research found a certain piece of evidence that adding 180 mg thiamine/kg DMI could increase the rumen pH and regulate the structure of the rumen microbial community *in vivo.* However, there is still limited experimental data on the effects of SARA on thiamine status, the damage to the structure of rumen epithelial cells, and the underlying mechanism of the epithelium alterations. For this purpose, a total of 18 Angus bulls (average 22.0-months-old) with an average live weight of 567.6 ± 27.4 kg were randomly allocated into a control treatment (CON), a high-concentrate diet treatment (HC), and a high-concentrate diet with the vitamin B1 supplement treatment (HCB). All bulls were conducted with a 7-day adjustment period followed by a 60-day-long main feeding procedure. Results indicated that ADFI and ADG significantly decreased in the HC treatment compared with CON (*P* < 0.05), while significantly increased after the VB1 supplement (*P* < 0.05). Besides, ruminal acetate content was significantly downregulated while propionate was significantly upregulated under the HC treatment compared with CON (*P* < 0.05); however, these alterations showed a completely inverse regulatory effect on the VB1 supplement compared with HC (*P* < 0.05). These changes causatively induced a significant decrease in the A/P ratio in the HC treatment compared with CON and HCB treatments (*P* < 0.05). Bacterial communities in the HC treatment could be separated from those in CON through PCoA axes 1 and 2. Meanwhile, the VB1 supplement significantly altered the bacterial communities compared with the HC treatment, except for HCB-3. Furthermore, the HC treatment significantly upregulated the expression of *JNK, Bax, Caspase-8, Caspase-3, Caspase-9*, and *Cyt-C* compared with CON, while significantly downregulated the expression of *Bcl-2*. The VB1 supplement showed a complete converse gene expression compared with HC. In conclusion, the VB1 supplement could effectively attenuate the alterations that occurred when exposed to high-concentrate diets, and help promote production performance through increased fermentability.

## Introduction

Subacute ruminal acidosis (SARA) becomes the most common nutritional metabolic disease in high-yielding dairy cows and the later fattening beef cattle because of the increasing consumption of high-concentrate diets in the modern feeding patterns (Xue et al., 2018b). SARA leads to serious damage to dairy cows and the fattening period of beef cattle. It is necessary to find a suitable mitigation method to attenuate SARA.

It is well-established that thiamine (VB1) is an essential cofactor required for carbohydrate metabolism, and ruminants have no dietary Vitamin B1 requirements because of the ability of the rumen microbes to synthesize extensive ruminal Vitamin B1 (Breves et al., 1981; Miller et al., 1986). Several previous studies have indicated that increasing non-fiber carbohydrates decreased the daily apparent synthesis of Vitamin B1 in the rumen (Schwab et al., 2006), and increased the duodenal flow of Vitamin B1 (Zinn et al., 1987). However, SARA caused by high-fermentable carbohydrates significantly altered the ruminal pH and microbial activity (Mao et al., 2015), which is likely to affect the amount of Vitamin B1 production. Our previous research found that adding 180 mg of Vitamin B1/kg DMI could increase the rumen pH and regulate the structure of the rumen microbial community *in vivo* (Wang et al., 2015; Pan et al., 2018). However, there is still limited experimental data on the effects of SARA on Vitamin B1 status, the damage on the structure of rumen epithelial cells, and the underlying mechanism of the epithelium alterations.

Rumen epithelium is stratified squamous epithelium (SSE) composed of stratum basale, stratum spinosum, stratum granulosum, and stratum corneum, which pivotally acted on ruminal micro-ecosystem homeostasis through regulating nutrient transportation and absorption, the barrier function, and microbial diversity (Stumpff et al., 2011; Zhang et al., 2013). Continuous exposure to acidic pH, induced by long-term feeding high-concentrate diet fortified ruminal osmotic pressure, invasively acted on rumen epithelial cells, and further induced cell viability decline, and even apoptosis (Tao et al., 2014; Gui and Shen, 2016; Chen et al., 2018).

Previous studies indicated that continuously exposed to acidic pressure induced by both lactate and volatile fatty acids (VFAs) caused the occurrence of rumen epithelial apoptosis (Luo et al., 2019), and promoted apoptosis-related genes expression, such as *Caspase-3, caspase-8, caspase-9, Bcl-2, Bax*, and *p53*, of rumen epithelial cells in both bovine and sheep (Gui and Shen, 2016; Dai et al., 2020). However, the underlying mechanism of acidity-induced apoptosis is still unclear.

Generally considered, the accumulation of reactive oxygen species (ROS) induced by acidic pH first activated JNKs through apoptosis signal-regulating kinase 1 (ASK1) and IRE1 (Zhang et al., 2010; Němcová-Fürstová et al., 2013). Following, the activated JNKs improved the expression of downstream apoptosis-related protein Caspase 3, and further upregulated the poly-ADP-ribose polymerase (PARP), while downregulated the Bcl-2 (Miao et al., 2018; Ren et al., 2020). Moreover, C/EBP homologous protein (CHOP) may also be activated by the JNK signaling pathway (Huang et al., 2019) to intensify endoplasmic reticulum mediated apoptosis by upregulating the expression of caspase-12 (Su et al., 2016). All these genes may synergistically express under acidic conditions and causatively induced cell apoptosis.

Therefore, in the present study, rumen microbiota responses to vitamin B1 supplements combined with the apoptosis-related genes that participated in the JNK signaling pathway were investigated to determine the underlying mechanism of rumen epithelial apoptosis induced by acidity. We hypothesized that continuous exposure to an acidity environment triggered the JNKs signaling pathway, and further upregulated the apoptosis-related genes to induce cell apoptosis.

## Materials and methods

### Experimental design

The experiment was conducted on the cattle farm of Yangxin Yiliyuan Halal Meat Co., LTD, Yangxin, Shandong, China. A total of 18 Angus bulls (average 22.0-months-old) with an average live weight of 567.6 ± 27.4 kg were randomly allocated into three treatments, which included CON: a control treatment, HC: a high-concentrate diet treatment, and HCB: a high-concentrate diet with the vitamin B1 supplement treatment. Each treatment contained six repeats, with one bull in each repeat. All bulls received a 7-day-long adjustment period followed by a 60-day-long main feeding procedure. All animals were reared according to the Chinese Guidelines for Animal Welfare, which was approved by the Animal Care and Use Committee of Jiangxi Agricultural University, with the approval number JXAULL-20220121. All bulls were distributed in an individual barn, which was separated into 18 hurdles, with one bull in each hurdle. All bulls were free to walk and adapt to water *ad libitum*.

Diets were formulated according to the National Standard for Beef Cattle to meet the requirement of bulls with 550–600 kg body weight. All bulls were fed two times per day at 06:00 and 18:00. Details of ingredient analysis and chemical composition of diets are shown in Table 1. A total of 180 mg/kg DM of Vitamin B1 (Vitamin B1 hydrochloride, purity ≥ 99%; Wanrong Science and Technology Development Co., Ltd., Wuhan, China) was added into the premix first and further provided for the bulls that received the HCB treatment.

**TABLE 1:** The experimental total mixed ratio of control treatment and high-concentration treatment.

Items	CON	HC
**Ingredients (% of DM)**		
Chinese wild rye	11.0	5.0
Corn silage	34.0	20.0
Alfalfa hay	15.0	15.0
Ground corn	10.0	30.0
Soybean meal, 43% CP	14.0	14.0
Cottonseed meal, 42% CP	5.0	5.0
DDGS, 27.5% CP	5.0	5.0
Whole cotton seed	3.0	3.0
Limestone meal	1.0	1.0
Calcium hydrogen phosphate	0.7	0.7
Sodium chloride	0.5	0.5
Premix^a^	0.8	0.8
**Nutrient composition (% of DM)**		
NE_L_^b^, MJ/kg	6.61	7.03
CP	18.16	18.10
Starch	19.95	30.82
NDF	36.18	27.61
ADF	23.43	17.72
NFC^c^	32.67	45.74
Ether extract	4.61	4.20
Ash	5.04	4.35
Calcium	0.88	0.84
Phosphorus	0.55	0.55

^a^Premix contained (per kg): 2,142 mg of Cu (as sulfate); 15,428 mg of Mn (as sulfate); 15,428 mg of Zn (as sulfate); 28 mg of Co (as chloride); 231 mg of I (as iodate); 57 mg of Se (as selenite); 2,285,000 IU of vitamin A; 457,000 IU of vitamin D; 11,400 mg of vitamin E. ^b^NE_L_ was estimated according to NRC (2001). ^c^NFC = 100–(%NDF + %CP + %ether extract + %ash) (NRC, 2001).

### Determination of productive performances

Average daily intake was shown as the dry matter intake (DMI) of each treatment, which was calculated through the deviation between the weight of the supply and the residue. The DMI of each treatment group (CON, HC, and HCB) was calculated based on the average performance of the six cattle. The body weight gain (BWG) of each bull was measured at the beginning and end of the trail, with an empty stomach in the morning, and average daily gain (ADG) was calculated as the ratio of BWG/days. Feed conversion ratio (FCR) was then calculated through the following equation:

F⁢C⁢R=A⁢D⁢F⁢I⁢(k⁢g)/B⁢W⁢G⁢(k⁢g)

### Ruminal fermentation parameters measurement

Rumen fluid samples of all 18 cows were collected on the last day through esophageal tubing for 3 h after morning feeding. The first tube of rumen fluid, which might be mixed with saliva, was discarded to ensure the purity of rumen microbiota. Each sample was divided into two portions. One was conducted to analyze ruminal pH [measured by Testo 206-pH1, Testo Instruments International (Shanghai) Co., Shanghai, China], rumen volatile fatty acids (VFAs, measured using a gas chromatograph, GC-2010, Shimadzu, Kyoto, Japan), and ammonia-N [NH_3_-N, determined by indophenol method and the absorbance value was measured through UV-2600 ultraviolet spectrophotometer (Shanghai Tianmei Scientific Instrument Co., LTD., Shanghai, China) at the 700 nm wavelength] (Xue et al., 2019). The other received part was first quick frozen by liquid nitrogen and then preserved at –80°C for further rumen microbial measurement.

### Rumen microbial community measurement

Rumen microbial DNA of each sample was first extracted using the Bacterial Genome DNA Extraction kit [DP302, Tiangen, Tiangen Biotech (Beijing) Co., Ltd]. Following this, the 16S rRNA gene V4 region was amplified using the universal primers 520F and 802R (Xue et al., 2018a). The Qiagen Gel Extraction kit (Qiagen, Hilden, Germany) was used to purify the mixture of PCR products, followed by the generation of sequencing libraries using the TruSeq^®^ DNA PCR-Free Sample Preparation kit (Illumina, USA). The Qubit@ 2.0 Fluorometer (Thermo Scientific) and the Agilent Bioanalyzer 2100 system were then applied for the assessment of the library quality. The Illumina HiSeq 4000 platform (Illumina Inc., San Diego, CA, USA) was used for the DNA sequencing process. High-quality clean tags were obtained according to the Quantitative Insights Into Microbial Ecology (QIIME, V1.7.0) quality controlling process, and sequences within similarity >97% were assigned to the same operational taxonomic unit (OTU). All following analyses included the α-diversity, β-diversity, and discrepancy bacterial community analyses were conducted based on the OTU results.

### Rumen epithelium sampling and apoptosis-related genes expression

The rumen endodermis epithelial samples were isolated immediately after slaughtering and quickly placed into the ice-cold PBS. The epithelial tissues were washed five times with ice-cold PBS, which is added with 0.5 mg/ml amphotericin B and 100 μg/ml gentamicin, followed by being cut into small pieces and quickly frozen into the liquid nitrogen.

Total RNA was extracted from each rumen epithelial sample using the RNA kit (Takara) according to the manufacturer’s instructions. The amount and quality of RNA were determined by a spectrophotometer (Bio-Rad Laboratories, Hercules, CA, USA). Total RNA of all samples was first inverse-transcripted into cDNA according to the instructions of transcript reverse transcriptase (Bio-Rad Laboratories, Hercules, CA, USA), and then quantified by an RT-PCR amplifier (Roche, Applied Science, Mannheim, Germany) soon after the primers designation. All primers were designed using the Primer 5.0 software according to the gene sequence provided by GenBank in NCBI and displayed in Table 2.

**TABLE 2:** PCR amplification primer design for apoptosis-related genes.

Name	Primer	Sequence	Size
GAPDH	Forward	5′-GTCGGAGTGAACGGATTTGG-3′	178 bp
	Reverse	5′-CGTTCTCTGCCTTGACTGTG-3′	
Caspase-3	Forward	5′-ACGGAAGCAAATCAGTGGAC-3′	167 bp
	Reverse	5′-GGTTTCCCTGAGGTTTGCTG-3′	
Caspase-8	Forward	5′-TCTGCTGCATCCTTACCCAT-3′	157 bp
	Reverse	5′-TCCCCTTGACAAGCCTGAAT-3′	
Caspase-9	Forward	5′-GTCCTGTGTCCGTTGAGAGA-3′	174 bp
	Reverse	5′-TCTCAGGGTTGCTATTGGGG-3′	
Bax	Forward	5′-AAGAAGCTGAGCGAGTGTCT-3	184 bp
	Reverse	5′-TGGCAAAGTAGAAAAGGGCG-3′	
Bcl-2	Forward	5′-TCTTTGAGTTCGGAGGGGTC-3′	162 bp
	Reverse	5′-GGCCATACAGCTCCACAAAG-3′	
JNK	Forward	5′-CGCTACTACAGAGCACCTGA-3′	210 bp
	Reverse	5′-GCACCCAACTGACCAAATGT-3′	
Cyt-C	Forward	5′-ATCGTCGCCCTGAAGTGTTA-3′	177 bp
	Reverse	5′-TTCGACATGGCCAACACATC-3′	

### Statistical analysis

Production performances and ruminal fermentation variables were first analyzed through a normal distribution test using the SAS procedure “proc univariate data = test normal,” and subsequently, the one-way ANOVA S-N-K test was received by SAS (SAS Institute, Inc., Cary, NC, USA). Significance would be considered when *P* < 0.05, while a tendency was considered when 0.05 ≤ *P* < 0.10. OTU abundances of each rumen bacteria were first conducted through a percentage transformation, and then a one-way ANOVA S-N-K test of SAS 9.2 was applied for the differential analysis. Alpha diversity and Beta diversity in our samples were calculated with QIIME 2 (Bolyen et al., 2018) and displayed with the R software (Version 3.3.1, R Core Team, Vienna, Austria). Principal coordinate analysis (PCoA) for different rumen methanogens was conducted using the R “vegan package.” Fold change of target gene expression was normalized by the housekeeping gene of GAPDH, and the discrepancies were determined by the 2^–ΔΔ*Ct*^ method.

## Results

### Production performances and rumen fermentability parameters

The production performances, including dry matter intake (DMI), average daily body weight gain (ADG), and the feed conversion ratio (FCR) were first calculated. Then, rumen fermentable parameters, including ruminal pH, volatile fatty acids (acetate, propionate, and butyrate), and ammonia-N were measured. These results are shown in Table 3.

**TABLE 3:** Effects of high-concentrate diet and thiamine supplementation feeding treatments on production and ruminal fermentability parameters (*n* = 6).

Items	Experimental treatments			SEM	*P*-value
	CON	HC	HCB		
ADFI (kg)	10.68^a^	9.07^b^	10.78^a^	0.278	0.014
ADG (kg)	1.32^a^	1.11^b^	1.47^a^	0.134	0.027
FCR	8.09^a^	8.17^a^	7.33^b^	0.263	0.031
Ruminal pH	6.45^a^	5.58^c^	6.12^b^	0.194	0.016
Acetate (mmol/L)	43.24^a^	42.62^b^	44.07^a^	0.273	0.038
Propionate (mmol/L)	15.89^b^	17.85^a^	14.84^c^	0.632	0.027
A/P	2.72^a^	2.39^b^	2.97^a^	0.213	0.017
Butyrate (mmol/L)	12.77	11.35	12.82	0.837	0.056
TVFA (mmol/L)	79.15	81.23	80.33	3.646	0.231
Ammonia-N (mg/100 ml)	10.49^b^	13.86^a^	11.37^b^	1.711	0.006

^a,b,c^Mean within a row with different letters differed significantly (P < 0.05); SEM, standard error of the mean. CON, control treatment; HC, high-concentrate diet treatment; HCB, high-concentrate diet supplemented with vitamin B1 treatment; ADG, average daily gain; ADFI, average daily feed intake; FCR, feed conversion ratio; A/P, acetate to propionate ratio; TVFA, total volatile fatty acid.

Specifically, ADFI and ADG significantly decreased in the HC treatment compared with CON (*P* < 0.05), while significantly increased after the VB1 supplement (*P* < 0.05), which causatively induced the significant decrease of FCR in the HCB treatment compared with CON and HC treatments (*P* < 0.05). When referred to the fermentable parameters, ruminal pH significantly declined under the HC treatment, while significantly increased after the VB1 supplement (*P* < 0.05). Besides, ruminal acetate content was significantly downregulated while the propionate was significantly upregulated under the HC treatment compared with CON (*P* < 0.05), while these alterations showed a completely inverse regulatory effect after the VB1 supplement compared with HC (*P* < 0.05). All these changes caused a significant decrease in the A/P ratio in the HC treatment compared with CON and HCB treatments (*P* < 0.05). On the contrary, the ammonia-N content significantly increased in the HC treatment compared with CON and HCB (*P* < 0.05). No other significant alterations were observed among all three treatments.

### Ruminal microbial communities measurement

In general, a total of 4,036 OTUs were obtained by performing OTU clustering on non-repetitive sequences according to 97% similarity. Taxonomic information indicated that a total of 18 phyla and 2,034 genera were identified after quality control, and it is displayed in Supplementary Table 1. All taxonomic bacteria were further applied for α-diversity, β-diversity, and differential communities’ investigation.

#### α-diversity

Alpha diversity was first investigated to determine the regulatory effects of HC and VB1 supplements on the complexity of rumen microbial diversity through Chao1, Shannon, Simpson, and ACE indexes. As shown in Table 4, the ACE index significantly decreased in the HC treatment compared with CON and HCB treatments (*P* < 0.05). No significant differences were found for the other indexes.

**TABLE 4:** Effects of small peptides supplement on the α-diversity of ruminal microbiota (*n* = 6).

Items	Experimental treatments			SEM	*P*-value
	CON	HC	HCB		
Shannon	8.29	8.14	8.23	0.15	0.182
Simpson	0.98	0.98	0.98	0.01	0.342
ACE	2263.4^a^	2156.5^b^	2266.4^a^	22.4	0.018
Chao1	2156.3	2131.4	2168.4	31.2	0.162

^a,b^Mean within a row with different letters differed significantly (*P* < 0.05). SEM, standard error of the mean.

CON, control treatment; HC, high-concentrate diet treatment; HCB, high-concentrate diet supplemented with vitamin B1 treatment.

#### β-diversity

**FIGURE 1:**
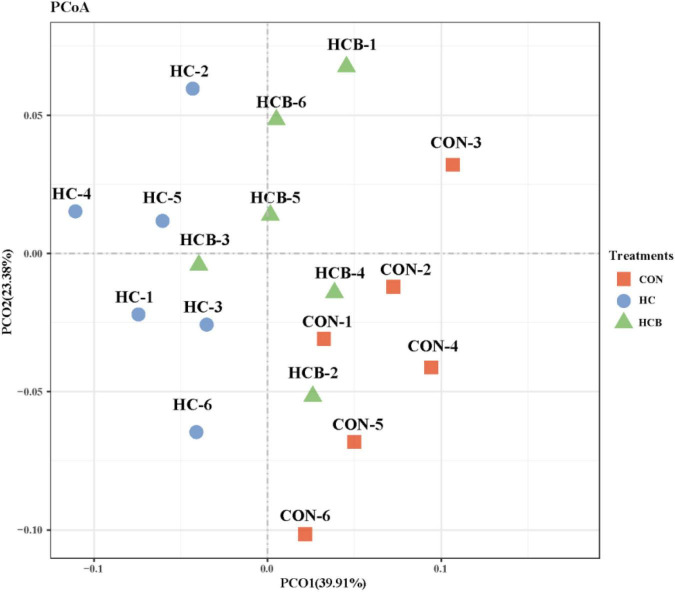
Principal coordinate analysis (PCoA) on community structures of the rumen microbiota between control treatment and small peptide supplement treatment. CON, control treatment; HC, high-concentrate diet treatment; HCB, high-concentrate diet supplemented with vitamin B1 treatment.

Differential analysis on rumen microbial communities among CON, HC, and HCB treatments was first proceeded through principal coordinates analysis (PCoA) to investigate the effects of HC and VB1 on the integrity of bacterial communities. As shown in Figure 1, PCoA axes 1 and 2 accounted for 39.91 and 23.38% of the total alteration, respectively. Bacterial communities in the HC treatment could be separated from those in CON through PCoA axes 1 and 2. VB1 supplement significantly altered the bacterial communities compared with the HC treatment, except for HCB-3.

Then, discrepancies in the relative abundance of bacteria community proceeded, and all the results are shown in Tables 5, 6.

**TABLE 5:** Effect of high-concentrate diet feeding and thiamine supplementation on relative abundance of rumen bacteria (%, level of phyla, *n* = 6).

Items	Experimental treatments			SEM	*P*-value
	CON	HC	HCB		
*Bacteroidetes*	54.78^a^	47.51^b^	49.91^ab^	3.274	0.011
*Firmicutes*	26.26^b^	35.57^a^	32.86^ab^	0.935	0.001
*Bacteria_noname*	6.34	5.32	5.89	1.459	0.202
*Proteobacteria*	7.06	6.83	6.25	0.769	0.169
*Fibrobacteres*	1.99^a^	0.64^b^	0.98^b^	0.258	0.028
*Spirochaetes*	0.95	1.00	0.72	0.066	0.187
*Actinobacteria*	0.42^b^	0.71^a^	0.60^a^	0.074	0.008
*Verrucomicrobia*	0.32	0.34	0.47	0.031	0.071
*Chlamydiae*	0.21^b^	0.59^a^	0.23^b^	0.059	0.001
*Cyanobacteria*	0.19^b^	0.14^b^	0.54^a^	0.156	0.001
*Planctomycetes*	0.10^b^	0.08^b^	0.16^a^	0.012	0.024
*Tenericutes*	0.08^b^	0.12^b^	0.19^a^	0.027	0.001
*Fusobacteria*	0.07	0.12	0.11	0.011	0.158
Others	1.23	1.03	1.09	0.016	0.002

^a,b^Mean within a row with different letters differed significantly (*P* < 0.05).

SEM, standard error of the mean. CON, control treatment; HC, high-concentrate diet treatment; HCB, high-concentrate diet supplemented with vitamin B1 treatment.

**TABLE 6:** Effect of high-concentrate diet feeding and thiamine supplementation on relative abundance of rumen bacteria (%, level of genera, *n* = 6).

Items	Experimental treatments			SEM	*P*-value
	CON	HC	HCB		
*g__Prevotella*	19.33	16.34	19.62	2.390	0.064
*g__Ruminococcaceae*	13.84^b^	15.70^a^	13.87^b^	1.290	0.005
*g__Succiniclasticum*	10.89	11.51	10.50	0.820	0.291
*g__Lachnospiraceae*	5.22	5.28	5.35	0.280	0.623
*g__Eubacterium*	4.84	4.97	5.01	0.210	0.141
*g__Rikenellaceae*	1.77^b^	3.14^a^	2.56^a^	0.250	0.015
*g__Ruminococcus*	3.77^b^	7.31^a^	5.64^a^	1.880	0.012
*g__Shuttleworthia*	2.72^a^	1.34^b^	2.13^ab^	0.520	0.032
*g__Prevotellaceae*	1.27	1.22	1.35	0.230	0.371
*g__Acetitomaculum*	1.28^a^	0.73^b^	1.33^a^	0.330	0.023
*g__Erysipelotrichaceae*	1.11	0.86	1.09	0.330	0.137
*g__Lachnoclostridium*	0.66	0.73	0.80	0.220	0.995
*g__Saccharofermentans*	0.63^a^	0.45^b^	0.64^a^	0.110	0.008
*g__Butyrivibrio*	0.48^b^	0.40^b^	0.61^a^	0.050	0.048
*g__Ruminiclostridium*	0.21	0.28	0.28	0.080	0.503
*g__Lachnospira*	0.32^a^	0.16^b^	0.23^b^	0.070	0.034
*g__Pseudobutyrivibrio*	0.11^b^	0.18^b^	0.33^a^	0.030	0.009
*g__Acidaminococcus*	0.13	0.07	0.21	0.120	0.167
*g__Selenomonas*	0.04^b^	0.11^a^	0.03^b^	0.040	0.001
*g__Lactobacillus*	0.05	0.06	0.08	0.050	0.936
*g__Pseudoramibacter*	0.04	0.02	0.03	0.030	0.089
*g__Bifidobacterium*	0.05^a^	0.03^b^	0.06^a^	0.010	0.027
*g__Escherichia-Shigella*	0.01	0.01	0.01	0.003	0.680
*g__Bacteroides*	0.01	0.02	0.01	0.010	0.571
*g__Succinivibrio*	0.03^a^	0.01^b^	0.03^a^	0.003	0.001
*g__Streptococcus*	0.01	0.01	0.01	0.006	0.083
*g__Butyricicoccus*	0.01	0.01	0.01	0.007	0.969
Others	31.17	29.05	28.21	1.450	0.211

^a,b^Mean within a row with different letters differed significantly (*P* < 0.05).

SEM, standard error of the mean. CON, control treatment; HC, high-concentrate diet treatment; HCB, high-concentrate diet supplemented with vitamin B1 treatment.

Discrepancies at the phyla level are displayed in Table 5. *Bacteroidetes* and *Firmicutes* contributed as the most two abundant bacteria communities, which accounted for 90% of total microbial profiles. The relative abundance of *Bacteroidetes* significantly decreased, while of *Firmicutes* significantly increased in the HC treatment compared with the CON (*P* < 0.05). Compared with HC, VB1 supplement slightly increased the *Bacteroidetes* while decreased *Firmicutes*, however, not significantly. In addition, the HC treatment significantly decreased the relative abundances of *Fibrobacteres*, while significantly increased *Actinobacteria* and *Chlamydiae* (*P* < 0.05). The VB1 supplement significantly increased *Cyanobacteria, Planctomycetes*, and *Tenericutes*, while significantly decreased *Chlamydiae* compared with the HC treatment (*P* < 0.05). No other significant alterations were observed.

Then, differential analyses of the relative abundances at the genera level were conducted and the results are shown in Table 5. *Prevotella, Ruminococcaceae*, and *Succiniclasticum* contributed as the three most abundant genera and accounted for nearly half of all microbiota profiles. Microbial communities included *Ruminococcaceae, Rikenellaceae, Ruminococcus*, and *Selenomonas* significantly proliferated, while the abundances of *Shuttleworthia, Saccharofermentans, Shuttleworthia, Acetitomaculum, Saccharofermentans, Lachnospira, Bifidobacterium*, and *Succinivibrio* significantly suppressed in the HC treatment compared with CON. Besides, the VB1 supplement significantly increased *Acetitomaculum, Saccharofermentans, Butyrivibrio, Pseudobutyrivibrio, Bifidobacterium*, and *Succinivibrio*, while significantly declined abundances of *Ruminococcaceae* and *Selenomonas* compared with HC (*P* < 0.05). No significant changes were found for other bacterial genera.

### Rumen epithelial apoptosis-related genes expression

**FIGURE 2:**
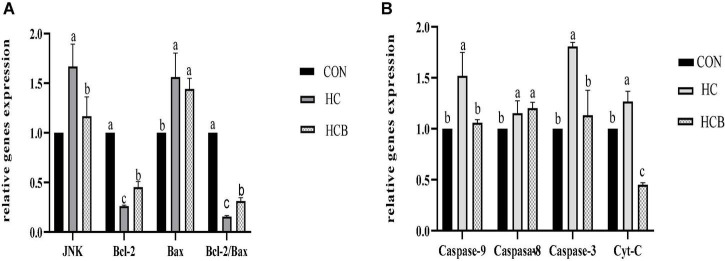
Effects of high-concentrate diets and vitamin B1 supplement treatments on the apoptosis-related genes expression of rumen epithelium. **(A)** Effects of high-concentrate diets and vitamin B1 supplement on the relative expression of JNK, Bcl-2, and Bax. **(B)** Effects of high-concentrate diets and vitamin B1 supplements on the relative expression of Caspase-9, Caspase-8, Caspase-3, and Cyt-C. CON, control treatment; HC, high-concentrate diet treatment; HCB, high-concentrate diet supplemented with vitamin B1 treatment. a,b,c means within different letters the gene expression differed significantly (*P* < 0.05).

The HC treatment significantly decreased the ruminal pH, which further caused epithelial cell apoptosis. Therefore, the apoptosis-related genes were detected and the results are shown in Figure 2. Compared with CON, HC treatment significantly upregulated the expression of *JNK, Bax, Caspase-8, Caspase-3, Caspase-9*, and *Cyt-C*, while significantly downregulated the expression of *Bcl-2*. The VB1 supplement remarkably decreased the expression of *JNK, Caspase-9, Caspase-3*, and *Cyt-C*, while significantly upregulated the expression of *Bcl-2*. Particularly, the ratio *Bcl-2/Bax* significantly decreased in HC compared with CON, while significantly increased after the VB1 supplement compared with the HC treatment.

### Interactive analysis

**FIGURE 3:**
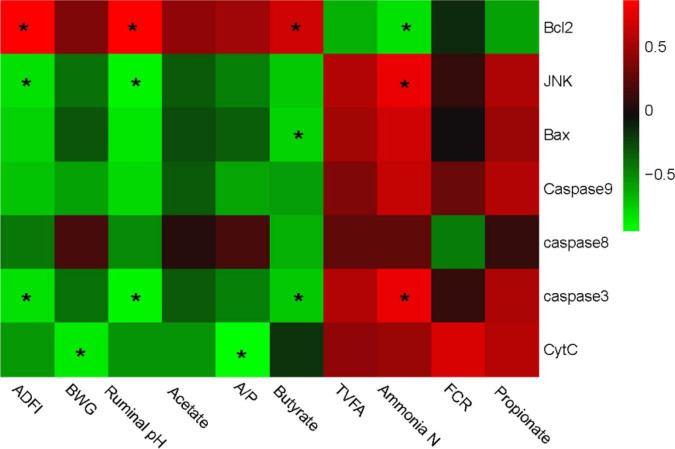
Correlation analysis between production performances, rumen fermentability parameters, and epithelial apoptosis-related genes expression. The red color represents a positive correlation while the green color represents a negative correlation. “*” means a significant correlation (| r| > 0.55, *P* < 0.05). ADFI, average daily feed intake, BWG, body weight gain; FCR, feed conversion ratio; TVFA, total volatile fatty acids.

Interactive analysis of the expression of apoptosis-related genes, productive performances, and the fermentability parameters are conducted and the result is shown in Figure 3.

Integrally, genotypic parameters could be separated into two clusters, based on the correlations with apoptosis-related genes. One consisted of ADFI, BWG, ruminal pH, acetate, and butyrate, which was positively correlated with *Bcl-2*, while negatively correlated with *JNK, Bax, Caspase-8, Caspase-3, Caspase-9*, and *Cyt-C*. The other cluster mainly consisted of TVFA, Ammonia-N, FCR, and propionate, which performed a completely contrary correlation with the apoptosis-related genes compared with the former one. Particularly, Bcl-2 gene expression was significantly positively correlated with ADFI, pH, and butyrate, while negatively correlated with ammonia-N content. JNK and caspase-3 showed a converse correlation compared with Bcl-2. Bax was negatively correlated with butyrate, while the cyt-C was negatively correlated with BWG and A/P. No other significant correlation was observed.

**FIGURE 4:**
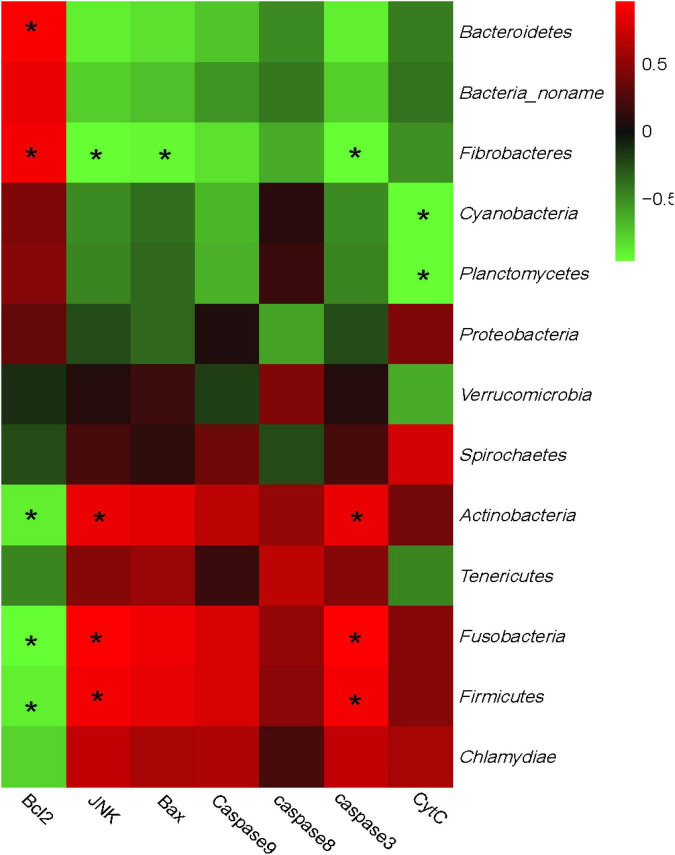
Correlation analysis between rumen bacteria community and epithelial apoptosis-related genes expression. The red color represents a positive correlation while the green color represents a negative correlation. “*” means a significant correlation (| r| > 0.55, *P* < 0.05).

Furthermore, a correlation analysis between the bacterial communities and the genes expression was conducted and the relationship is displayed in Figure 4. Bacteria could also be separated into two big clusters based on their relationship with gene expressions. One mainly consisted of *Bacteroidetes, Fibrobacteres, Cyanobacteria*, and *Planctomycetes*, and shows a positive correlation with *Bcl-2*, while negatively correlated with *JNK, Bax, Caspase-8, Caspase-3, Caspase-9*, and *Cyt-C*. Meanwhile, the other cluster mainly consisted of *Firmicutes, Actinobacteria*, and *Chlamydiae* and showed a complete converse correlation with the apoptosis-related genes compared with the former cluster. Specifically, the expression of *Bcl-2* was positively correlated with *Bacteroidetes* and *Fibrobacteres*, while negatively correlated with *Firmicutes* and *Actinobacteria.* Similar to the correlation with phenotypic parameters, the expressions of JNK and caspase-3 also showed a complete correlation with *Bcl-2.*

## Discussion

Subacute ruminal acidosis epidemically occurs among high-yielding dairy cows and later fattening beef cattle as a consequence of feeding higher grain diets (Plaizier et al., 2008). Vitamin B1 functionally participated in the physiological carbohydrate metabolism to regulate the potential metabolic disorders during SARA. However, the potential mechanism of VB1 on rumen epithelial function and cell transportation is still unclear. We systematically summarized the effects of VB1 on the rumen epithelial apoptosis process, and interpret the underlying attenuating mechanism of VB1 on SARA.

### Effects of VB1 on rumen fermentability

Rumen fermentability determined the nutritional provision for body condition, while Vitamin B1 supplement significantly promoted the fermentability, including the enhancement content of acetate, propionate, and butyrate.

Ruminal infusion of Vitamin B1 is supposed to be an effective strategy to alleviate SARA in our study, as it helps in increasing the ruminal pH, reducing the ruminal lactate concentration, and improving the component of total VFA. The mechanism of Vitamin B1 on ruminal fermentation is related to its effects on microbes in the rumen. Wang et al. (2015) reported that Vitamin B1 supplementation significantly reduces the population of *Streptococcus bovis* (lactate-producing bacteria) and increases the population of *Megasphaera elsdenii* (lactate utilizers and convert ruminal lactate into propionate). Besides, a higher Vitamin B1 concentration in the rumen can support the protozoa population (Höltershinken et al., 2003); the increasing protozoa could store fermentable carbohydrates for a short time and consequently help to stabilize ruminal pH. Moreover, Vitamin B1 supplementation enhanced the activity of PDH and α-KGDHC, and consequently, increased the amount of pyruvate flow to the TCA cycle and reduced the conversion of pyruvate to lactate.

Specifically, the acetate concentration showed a high positive correlation to the Vitamin B1 concentration, this result was mainly attributed to the formation process of acetate. The major portion of the acetate is derived from the decarboxylation of pyruvate, which was degraded from structural carbohydrates (Baldwin et al., 1963). The decreased dietary structural carbohydrates and blocked pyruvate decarboxylation caused by Vitamin B1 deficiency in SARA cows inhibited the acetate generation and also lowered the ruminal acetate concentration.

Propionate is generally produced by succinate or from lactate *via* the acrylate pathway. The contribution of the acrylate pathway traditionally increased accompanied by higher carbohydrate availability (Baldwin et al., 1963). In our study, a high proportion of grain in the SARA diet could be rapidly fermented into large amounts of pyruvate, and then converted into propionate through the generating bacteria (Van Soest, 1994). Vitamin B1 deficiency under high-concentrate levels restricted the regular metabolism of pyruvate into acetyl-CoA, and may further lead to the accumulation of propionate. In the present study, vitamin B1 content significantly decreased, which interpreted the higher propionate concentration in the rumen.

### Effects of VB1 on rumen epithelial functions

Rumen epithelial cell viability represented the underlying functions of nutrient absorption, the fundamental for bioactivities transportation, and the barrier functions. The viability was impacted by certain factors, including physical damages, such as pressure, and chemical injuries, such as the acidity treatment (Luo et al., 2019). When ruminants suffered SARA, the lower ruminal pH induced certain syndromes to threaten the homeostasis of the rumen micro-ecosystem (Mu et al., 2021). The increased hydrogen ions fortified ruminal osmotic pressure, which further aggressively impact the regular ion exchange process that occurred in epithelial cells and causatively induced decreased cell viability.

Simultaneously, alterations of the rumen microbiome generated unconventional metabolites, such as biogenic amines and lipopolysaccharide, which further induced inflammatory responses, suppressed cell viability, and disrupted the functions of epithelial cells (Wang et al., 2021). Apart from causing serious inflammatory responses, cellular anti-oxidative capacity is also a noticeable factor that impacts cell viability. The reactive oxygen scavenging ability ensured the normal cellular nutrients transportation. However, the previous study shows that the accumulation of reactive oxygen species (ROS) induced by acidic pH significantly restrained cell viability (Schmidt et al., 2021), which ultimately stimulated cell apoptosis.

Generally, apoptosis occurs when cells receive stress, invasive damage, and gene regulation. The rumen epithelial cell apoptosis process was stimulated when homeostasis was disturbed, as reported by Ma et al. (2021). The acidity treatment first promoted ROS-dependent intrinsic cell apoptosis by activating a JNK signaling pathway (Jiang et al., 2017). Indeed, in our present study, ROS levels, as well as the JNK gene and protein expression, significantly upregulated, which further confirmed the inductive effects of JNK signaling pathways. JNK signaling pathway executed an efficient function in cell apoptosis through c-Jun phosphorylation (Dhanasekaran and Reddy, 2008). The significant upregulation of JNK phosphorylation, as shown in the western blotting, further convincedly proves the inductive effects of JNK on cell apoptosis.

### Underlying mechanism of the acidity treatment on apoptosis

Traditionally, cell apoptosis was primarily induced by the upregulation of the *Caspase 3* gene expression, which could be stimulated through both mitochondrial and nucleonic pathways. JNK induced the cleavage of *Bid*, inhibited the anti-apoptotic proteins, such as *Bcl2*-family apoptotic proteins, and activated the expression of pro-apoptotic genes, such as *Bax* (Bcl2-associated protein). The activated *Bax* forms membrane channels through which apoptogenic proteins, such as cytochrome C, can be released (Ansari et al., 2021). In our research, the acidity treatment significantly up-regulated the expression of *Bax* and *cyt-C*, and down-regulated of the expression *Bcl2*. These findings showed a specific confirmation of the occurrence of mitochondrial apoptosis in an acidic environment.

The mitochondria-induced apoptosis is the most common process in the extrinsic pathway, of which the crucial event is the release of cytochrome C from the inner membrane space of mitochondria. Then, the released cytochrome C formed the apoptosomes combined with caspase-9, which activate the caspase-9 cascade process and finally triggered the epithelial cell apoptosis (Araya et al., 2021).

**FIGURE 5:**
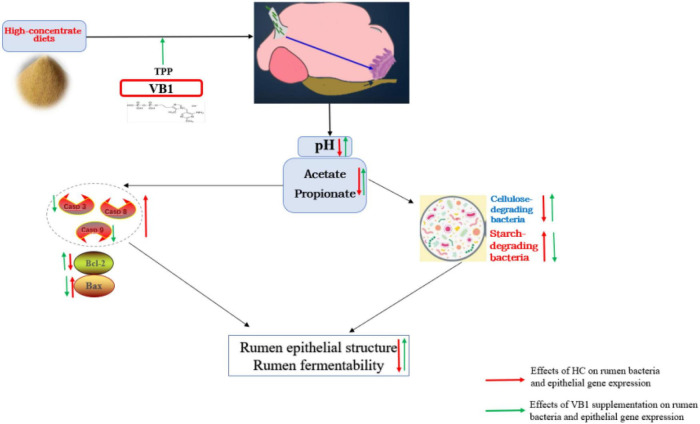
Potential attenuation mechanism scheme of Vitamin B1 on high-concentrate diets inducing rumen disorders.

In conclusion, VB1 supplement could effectively attenuate metabolic disorders, and the potential attenuation mechanism scheme just as shown in Figure 5. VB1 supplement promote epithelial function by alleviating cell apoptosis when exposed to high-concentrate diets, and finally promoted production performance.

## Data availability statement

The original contributions presented in the study. are publicly available. This data can be found here: NCBI SRA database, accession number PRJNA644053.

## Ethics statement

The animal study was reviewed and approved by JXAULL-20220121.

## Author contributions

LY and FX designed the study. PM, CS, ML, YaS, and YK conducted the experiment. CS, HY, and PYM contributed to the manuscript writing and English editing. ZY and YuS contributed to parameter measurement and data analysis. All authors contributed to the article, approved the submitted version, carefully read the manuscript, and were accountable for all aspects of the work.

## Supplementary material

Supplementary Table 1 Taxonomy results of each sample.
